# Methicillin-Resistant *Staphylococcus aureus* (MRSA) and Vancomycin-Resistant Enterococci (VRE) in Nosocomial Infections: A Systematic Review of Resistance, Pathogenesis, and Clinical Management

**DOI:** 10.3390/microorganisms14020428

**Published:** 2026-02-11

**Authors:** Lucian-Daniel Peptine, Andreea-Eliza Zaharia, Nicoleta-Maricica Maftei, Cosmin-Răducu Răileanu, Elena-Roxana Matache (Vasilache), Alice-Crina Conea, Bianca-Ioana Chesaru, Dana Tutunaru, Oana-Maria Dragostin, Liliana Mititelu-Tarţău, Gabriela Gurău

**Affiliations:** 1 Dunarea de Jos University of Galati, Faculty of Medicine and Pharmacy, Research Centre in the Medical-Pharmaceutical Field, 800008 Galati, Romania; lucian.peptine@ugal.ro (L.-D.P.); andreea.zaharia@ugal.ro (A.-E.Z.); elena.matache@ugal.ro (E.-R.M.); dr.alice.crina@gmail.com (A.-C.C.); bchesaru@ugal.ro (B.-I.C.); dana.tutunaru@ugal.ro (D.T.); oana.dragostin@ugal.ro (O.-M.D.); gabriela.gurau@ugal.ro (G.G.); 2 Sf. Ioan Emergency Clinical Hospital for Children, 80008 Galati, Romania; 3 Sf. Apostol Andrei County Emergency Clinical Hospital, 80008 Galati, Romania; 4 Grigore T. Popa University of Medicine and Pharmacy, 700115 Iasi, Romania; lylytartau@yahoo.com

**Keywords:** MRSA, VRE, methicillin resistance, vancomycin resistance (*vanA*/*vanB*), biofilm, rapid molecular diagnostics, PBP2a, AUC/MIC, infection prevention and control, antimicrobial stewardship

## Abstract

*Staphylococcus aureus* (MRSA) and vancomycin-resistant enterococci (VRE) are high-burden healthcare-associated pathogens that increase mortality, prolong hospitalisation, and drive substantial healthcare costs worldwide. These infections are associated with high morbidity, increased mortality, prolonged hospital stays, and significant costs, particularly among immunocompromised patients or those with extended hospitalizations. This systematic review was conducted and reported in accordance with PRISMA 2020, aiming to synthesise existing data on the epidemiology, resistance mechanisms, clinical manifestations, and strategies for the diagnosis, treatment, and prevention of MRSA and VRE infections. Data were qualitatively synthesised. A total of 113 records published between 2020 and 2025 met the inclusion criteria and were identified through searches in multiple bibliographic databases and publisher platforms (e.g., PubMed, Scopus, Web of Science). MRSA and VRE are implicated in numerous severe infections, including ventilator-associated pneumonia, catheter-associated urinary tract infections, endocarditis, and bacteraemia. Antimicrobial resistance is driven by the *mecA*, *vanA*, and *vanB* genes, while biofilm formation further complicates therapeutic efforts. Biofilm formation can promote antibiotic tolerance (slower killing without an increase in MIC) and persistence (survival of ‘persister’ cells), distinct from genetic resistance, and may complicate therapy in selected infections. Effective strategies include appropriate anti-MRSA/anti-VRE agents (e.g., ceftaroline for MRSA; linezolid or daptomycin for VRE), active screening, stringent infection prevention and control measures, and antimicrobial stewardship programmes. Implementation is often hindered by institutional barriers, limited resources, and insufficient staff training. A multidisciplinary, evidence-based approach is essential for the effective management of these infections. Reducing this burden requires coordinated implementation of rapid diagnostics, stringent infection prevention and control, and antimicrobial stewardship, supported by sustained institutional and public health investment.

## 1. Introduction

Nosocomial infections, also referred to as healthcare-associated infections (HAIs), represent a major challenge for health systems worldwide [1]. They contribute to prolonged hospital stays, increased healthcare costs, elevated morbidity and mortality rates, and significantly to the escalation of antimicrobial resistance [2]. While a wide variety of pathogens can be implicated in HAIs, Gram-positive cocci, particularly *Staphylococcus aureus* (*S. aureus*) and *Enterococcus* species, play a central role in the aetiology of the most severe and difficult-to-treat hospital-acquired infections [3]. Although Gram-negative pathogens also contribute substantially to HAIs, this systematic review intentionally focuses on multidrug-resistant Gram-positive cocci—specifically Methicillin-resistant *S. aureus* (MRSA) and vancomycin-resistant enterococci (VRE)—because of their distinct resistance determinants and the targeted prevention and clinical management strategies they require.

Among these Gram-positive cocci, *S. aureus* and *Enterococcus faecium*/*faecalis* are of particular concern due to their ability to rapidly acquire and disseminate antibiotic resistance [4]. MRSA remains one of the leading causes of nosocomial infections globally, implicated in pneumonia, bloodstream infections, surgical wound infections, and endocarditis [5]. Similarly problematic are VRE, particularly *E. faecium* and *E. faecalis*, which are frequently associated with catheter-associated urinary tract infections, bacteraemia, and intra-abdominal infections. The presence of resistance genes such as *mecA*, *vanA*, and *vanB* severely limits therapeutic options and significantly complicates infection control efforts [6].

The clinical impact of MRSA and VRE is further exacerbated by their capacity to form biofilms, survive under adverse environmental conditions, and persist on medical surfaces and equipment [7]. 

These pathogens are not only difficult to eradicate once established within a healthcare facility, but they also possess a remarkable ability to transfer resistance genes horizontally, facilitating rapid spread within healthcare environments [8]. Despite the implementation of infection control programmes and antibiotic stewardship initiatives, MRSA and VRE continue to pose a major threat in both high-resource hospitals and those located in resource-limited settings [9]. 

Although the literature on MRSA and VRE is extensive, most studies address isolated aspects (e.g., resistance mechanisms, virulence, or clinical outcomes), and an up-to-date integrative synthesis linking resistance, pathogenesis, epidemiology, and clinical management is still lacking [10]. This review article aims to address this gap by providing a comprehensive update on the molecular basis of resistance in MRSA and VRE, epidemiological trends, virulence factors, diagnostic challenges, therapeutic strategies, and infection prevention and control measures.

The primary objective of this paper is to critically assess the current state of knowledge regarding MRSA and VRE in the context of nosocomial infections, to highlight unresolved clinical and microbiological challenges, and to outline future directions for research and intervention. By consolidating recent findings from the scientific literature, we aim to support clinicians, microbiologists, and policymakers in the development of more effective diagnostic, therapeutic, and preventive strategies against these multidrug-resistant Gram-positive pathogens.

### 1.1. Epidemiology of MRSA and VRE in Nosocomial Settings

The epidemiology of MRSA and VRE in nosocomial infections is dynamic and heterogeneous, shaped by regional variation, local outbreaks, and differences across healthcare settings. Globally, MRSA is reported across all regions, with higher prevalence in North America, Eastern Europe, and Southeast Asia, whereas VRE was initially most prevalent in North America and Western Europe; more recent data indicate increasing VRE incidence in oncology and intensive care units (ICUs) in additional regions [11]. In Europe, approximately 20% of *S. aureus* isolates are MRSA, while in U.S. hospitals the proportion ranges from 33% to 55% [12]. Point-prevalence surveys across European hospitals show variable HAI rates, yet MRSA often represents a substantial proportion of *S. aureus* cases [13]. Overall, MRSA proportions are generally higher than VRE, but VRE can reach clinically significant levels in high-risk units, particularly transplant and haematology services [14]. Selected reported proportions/prevalence estimates of MRSA and VRE in nosocomial settings are summarised in Table 1.

**Table 1:** Selected reported proportions/prevalence estimates of MRSA and VRE in nosocomial settings (as cited in this section).

Context (Region/Setting)	MRSA (Reported Proportion)	VRE (Reported Proportion/Prevalence)	Ref.
Europe—*S. aureus* isolates	20%	-	[12]
U.S. hospitals—*S. aureus* isolates	33–55%	-	[12]
Europe—nosocomial *S. aureus* infections	5–25%; >30% in high-risk centres	-	[15]
Multi-hospital study (169 hospitals)	-	4.8% (*E. faecium*), 0.3% (*E. faecalis*); ICU: 0.1–0.7%	[14]
Transplant/haematology centres	-	up to 15–20%	[14]

In hospitals, MRSA commonly causes postoperative wound infections, ventilator-associated pneumonia, and bacteraemia, especially in surgical wards and ICUs [15]. VRE is particularly associated with catheter-associated urinary tract infections, bacteraemia, and intra-abdominal infections, with higher prevalence reported in oncology, haematology, and transplant wards [14].

Transmission is primarily contact-mediated: the hands of healthcare workers remain the dominant vector when hand hygiene is suboptimal [16], and contaminated high-touch surfaces and shared medical equipment further amplify spread [17,18]. Asymptomatically colonised patients (nasal carriage of *S. aureus* and intestinal carriage of enterococci) serve as reservoirs [19]; notably, VRE transfer from patient hands to surfaces appears substantially more likely than the reverse, underscoring the primacy of hand hygiene [20]. Outbreaks may also arise from atypical sources (e.g., contaminated devices at manufacture, water/cleaning solutions, or failures in ventilation/drainage systems), requiring rigorous epidemiological investigation [21]. Collectively, these data support continuous surveillance, targeted screening in high-risk units, and strengthened infection prevention and control measures to interrupt transmission and reduce the clinical and economic burden of MRSA and VRE.

### 1.2. Mechanisms of Antimicrobial Resistance

Methicillin resistance in *S. aureus* is primarily mediated by the *mecA* gene, which encodes an alternative penicillin-binding protein, PBP2a, with low affinity for most β-lactam antibiotics. This allows MRSA to survive treatment with methicillin, oxacillin, and other commonly used penicillins [22]. PBP2a enables the bacterium to continue cell wall synthesis even in the presence of inhibitory concentrations of β-lactams. The *mecA* gene is carried on the staphylococcal cassette chromosome mec (SCCmec), a mobile genetic element whose mobility and structural diversity facilitate dissemination among staphylococcal populations, contributing to the diversification of MRSA lineages and the emergence of multidrug-resistant strains [23]. 

In *Enterococcus* spp., vancomycin resistance is primarily mediated by the *vanA* or *vanB* gene clusters, which remodel the D-Ala–D-Ala target to D-Ala–D-Lac, markedly lowering vancomycin binding and efficacy [24]. The *vanA* operon typically confers high-level resistance to both vancomycin and teicoplanin (*VanA* phenotype) and is commonly borne on transferable plasmids and transposons, facilitating rapid dissemination in hospital environments. In contrast, *vanB* confers variable resistance to vancomycin while generally remaining susceptible to teicoplanin; it is also mobile and more frequently observed in specific geographic regions or in relation to certain clinical practices [25]. Other van operons (e.g., *vanC*/*E*/*G*) are associated with alternative modifications such as D-Ala-D-Ser.

In addition to *mecA*, *vanA*, and *vanB*, both MRSA and VRE can acquire additional resistance genes, often located on plasmids or integrons, which encode antibiotic-inactivating enzymes (e.g., β-lactamases, aminoglycoside-modifying enzymes), efflux pumps, target site modifications, or alterations in cell permeability [26]. The co-localisation of multiple resistance genes on mobile genetic elements significantly increases treatment complexity and complicates epidemiological control.

Understanding these molecular mechanisms is not merely academic; it is essential for the development of rapid diagnostic tests, the potential use of resistance markers in screening, and the guidance of targeted therapy. Moreover, knowledge of the underlying genetic basis of resistance can support the development of novel antibiotics that circumvent resistance mechanisms or molecules capable of inhibiting specific resistance genes or proteins.

**Figure 1:**
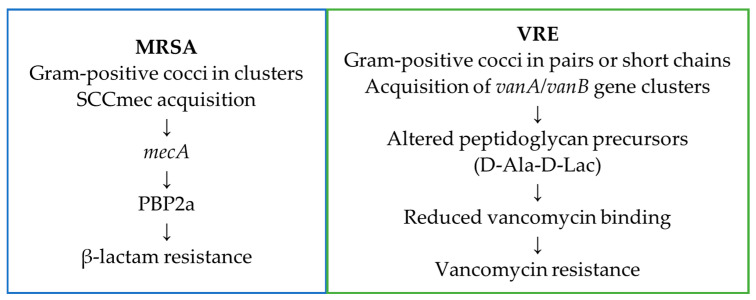
Simplified schematic representation of methicillin resistance in MRSA and vancomycin resistance in VRE. In MRSA, acquisition of the SCCmec element carrying *mecA* results in the expression of PBP2a and resistance to most β-lactam antibiotics. In VRE, *vanA* or *vanB* gene clusters result in the synthesis of altered peptidoglycan precursors terminating in D-Ala-D-Lac, thereby reducing vancomycin binding and conferring vancomycin resistance.

To enhance clarity and support the understanding of resistance dissemination, Figure 1 schematically illustrates the key mobile genetic elements involved in antimicrobial resistance in MRSA and VRE.

### 1.3. Pathogenesis and Virulence Factors

The virulence factors of MRSA and VRE play essential roles in their capacity to invade tissues, survive in hostile environments, and cause severe infections. These include a wide array of toxins, adhesion proteins, biofilm-forming mechanisms, and factors that enable colonisation and immune evasion.

*S. aureus*, including MRSA strains, produces a variety of cytotoxic toxins (α-, β-, γ-, and δ-toxins), as well as proteins involved in host cell destruction and immune evasion, such as leukocidins and exfoliatins [27]. These toxins contribute to cellular damage, leukocyte infiltration, and tissue necrosis, manifesting clinically as abscesses, severe pneumonia, and skin infections. The ability to form biofilms, facilitated by adhesion proteins that bind fibrinogen and fibronectin (MSCRAMMs), protects bacteria from both antibiotic action and the host immune system, allowing them to persist on catheters, prosthetic devices, and other medical equipment [28].

There are marked differences between community-acquired MRSA (CA-MRSA) and hospital-acquired MRSA (HA-MRSA) strains, which affect both pathogenic potential and epidemiology. CA-MRSA strains, such as USA300, often produce Panton-Valentine leukocidin (PVL), a toxin associated with increased virulence and linked to cases of necrotising pneumonia and aggressive skin infections in otherwise healthy individuals [29]. These strains typically carry smaller SCCmec elements and are resistant to fewer antibiotic classes, whereas HA-MRSA strains harbour larger SCCmec elements, conferring multi-drug resistance, and are predominantly associated with infections in hospitalised patients with comorbidities or following invasive procedures [30].

In enterococci, particularly VRE species (*E. faecium* and *E. faecalis*), pathogenesis is linked to their ability to colonise the gastrointestinal tract and subsequently translocate into the bloodstream or tissues, leading to bacteraemia or intra-abdominal infections [31]. Biofilms formed on mucosal surfaces or medical devices shield the bacteria and enable survival in hostile environments. Enzymes such as gelatinase and serine proteases, along with surface-associated aggregation proteins (e.g., Ebp pili), contribute to tissue adherence and invasion [32]. Furthermore, enterococci have a remarkable ability to horizontally transfer resistance genes via plasmids and transposons, thereby amplifying the spread of aggressive and resistant phenotypes [33].

These pathogenetic processes reflect a remarkable degree of biological adaptability, underpinned by biofilm formation, toxin production, interaction with the host immune system, and genetic plasticity. Understanding these mechanisms is important not only for developing effective therapies but also for designing preventive strategies that target adherence and colonisation pathways.

### 1.4. Clinical Manifestations of MRSA and VRE Infections

Infections caused by MRSA and VRE present a wide spectrum of clinical manifestations, ranging from localised conditions to severe systemic infections, often with specific features in immunocompromised patients.

Pneumonia, particularly in intensive care settings, often leads to severe clinical deterioration. Ventilator-associated pneumonia (VAP) is frequently linked to MRSA and presents with fever, purulent secretions, radiographic infiltrates, and rapid respiratory failure, often requiring intensive mechanical ventilation [34].

Post-surgical wound infections are common in preoperatively colonised MRSA patients, typically manifesting as local erythema, purulent discharge, and potential progression to sepsis, sometimes necessitating reoperation and combined antibiotic therapy [35].

Urinary tract infections (UTIs) caused by MRSA or VRE are frequently observed in patients with long-term urinary catheterisation. Clinical signs include dysuria, fever, urinary symptoms, and a high risk of secondary bacteraemia. Particularly dangerous in long-term hospitalised or immunocompromised patients, such infections may progress rapidly to sepsis [36].

Endocarditis caused by MRSA or VRE represents a severe clinical form with high mortality rates. MRSA is responsible for approximately 20–25% of nosocomial endocarditis cases. Clinical features include persistent fever, cardiac insufficiency, septic emboli, and valvular vegetations detected via echocardiography [37]. *Enterococcus*, especially resistant *E. faecalis*, is often involved in subacute endocarditis, with progressive onset and potential for embolic complications [38].

Immunocompromised patients, particularly those with cancer, organ transplants, hepatic disorders, or those undergoing dialysis, are especially vulnerable. In such patients, MRSA and VRE infections may progress fulminantly, manifesting as severe sepsis, multiple infection sites, and requiring intensive care support. In VRE cases, intestinal colonisation facilitates bacterial translocation into the bloodstream, often leading to serious intra-abdominal complications [39]. Compromised immunity or neutropenia further increases risk, and prognosis is frequently poor if the infection is not promptly diagnosed and appropriately treated.

The wide clinical variability from minor skin infections to severe conditions such as necrotising pneumonia, sepsis, and endocarditis necessitates a multidisciplinary approach and rapid diagnosis to initiate targeted therapy. In immunocompromised patients, early detection and close monitoring are essential to improve outcomes and prevent fatal complications.

## 2. Materials and Methods

PRISMA statement and registration. This systematic review was conducted and reported in accordance with the PRISMA 2020 statement. The review protocol was not registered (e.g., PROSPERO, York, UK), and no protocol amendments were made.

Information sources and search strategy. We performed a systematic search in bibliographic databases and publisher platforms (PubMed, Scopus, Web of Science, and relevant publisher sites including ScienceDirect, SpringerLink, Wiley Online Library, and Frontiers), covering publications from 1 January 2020 to 31 January 2025; the last search was run on 30 September 2025. The full search strategy is provided in Appendix A, Table A1.

**Figure 2:**
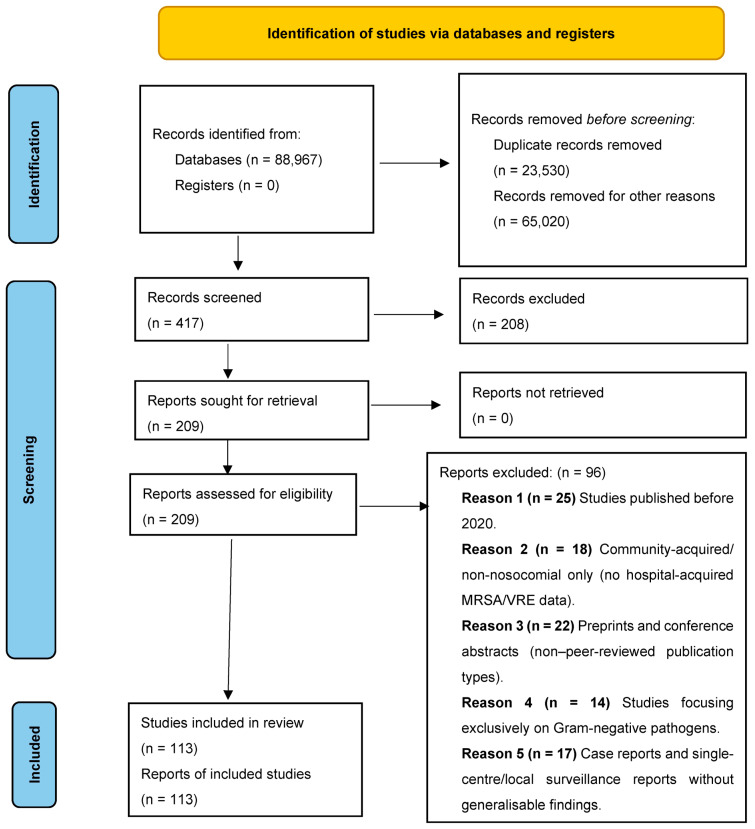
PRISMA Flow Diagram illustrating the study selection process for MRSA- and VRE-related nosocomial infection prevention, diagnosis, and treatment studies published between January 2020 and September 2025 (last search: 30 September 2025).

Study selection and synthesis. Records were screened by title/abstract and then assessed in full text for eligibility (see Section 2.2). A total of 113 studies met the inclusion criteria (Figure 2). Due to heterogeneity in study designs, populations, and outcomes, evidence was synthesised qualitatively using a structured narrative synthesis approach. In addition to the included studies, a small number of recent publications outside the predefined eligibility criteria (e.g., One Health/veterinary or experimental models) were cited to provide contextual background and were not part of the PRISMA-counted evidence synthesis.

### 2.1. Objectives and Research Question

The research question was: What is the impact of MRSA- and VRE-associated nosocomial infections on clinical practice, and what strategies are most effective for prevention, diagnosis, and management in hospital settings? We systematically identified and qualitatively synthesised evidence on epidemiology, resistance mechanisms, clinical manifestations, diagnostics, treatment, and infection prevention and control, with the aim of highlighting actionable gaps and priorities for future research.

### 2.2. Identification of Relevant Articles

The detailed search strategy (including database-specific syntax, controlled vocabulary where applicable, and Boolean operators) is provided in Appendix A (Table A1). The search covered 2020–2025, with the last search run on 30 September 2025 (Section 2). In addition to database searching, we performed reference list screening of eligible studies and relevant reviews (backward citation tracking) and forward citation tracking using database tools (e.g., “Cited by”) where available.

Eligibility criteria. We included peer-reviewed, English-language publications. Preprints were not included in the formal evidence synthesis; where cited, they were used only to contextualise emerging data. We included records relevant to MRSA and/or VRE in healthcare-associated (hospital) infections, addressing epidemiology, resistance mechanisms, pathogenesis/virulence, clinical manifestations, diagnostics, treatment, and infection prevention and control. Eligible study designs included clinical trials, observational studies, molecular/microbiological studies, and evidence syntheses (systematic reviews and meta-analyses). We excluded editorials, letters, and other opinion pieces, as well as studies lacking direct applicability to MRSA/VRE nosocomial infections (e.g., studies restricted to susceptible strains or non-healthcare settings without clear clinical relevance).

### 2.3. Eligibility Criteria for Study Selection

#### 2.3.1. Inclusion Criteria

Records were eligible if they addressed nosocomial or healthcare-associated infections caused by MRSA and/or VRE and contributed clinically relevant information on one or more of the following:

- Epidemiology and burden across clinical settings (e.g., ICU, oncology, surgery, geriatrics);
- Molecular mechanisms of antimicrobial resistance (e.g., *mecA*/*mecC*; *vanA*/*vanB* and related determinants);
- Clinical manifestations, severity, complications, and outcomes;
- Diagnostic strategies, including conventional microbiology and rapid molecular methods;
- Therapeutic options and challenges for multidrug-resistant infections;
- Infection prevention and control measures and antimicrobial stewardship initiatives.

Eligible publication types included original research (clinical, observational, and molecular/microbiological studies), systematic reviews, meta-analyses, and relevant practice guidelines. Peer-reviewed English-language publications were prioritised. Preprints (bioRxiv/medRxiv) were not included in the formal evidence synthesis (see Section 2.2).

#### 2.3.2. Exclusion Criteria

We excluded:

- Single case reports lacking broader relevance or sufficient data on prevalence, management, or control of MRSA/VRE;
- Studies focusing on community-acquired infections with susceptible *S. aureus* or non-VRE *Enterococcus* spp. without clear applicability to nosocomial infections;
- Studies focused exclusively on the development of new antibiotics without linkage to clinical or epidemiological hospital context;
- Editorials, letters, commentaries, and other opinion pieces;
- Records lacking direct clinical relevance to MRSA/VRE healthcare-associated infections.

#### 2.3.3. Screening Process and Inclusion Decisions

Study selection was conducted in two stages by two reviewers working independently: (i) title and abstract screening and (ii) full-text assessment against the predefined eligibility criteria. Discrepancies were resolved through discussion and consensus. The study selection process is summarised in the PRISMA flow diagram (Figure 2).

#### 2.3.4. Data Collection Methods

Data extraction was performed using a standardised Microsoft Excel form to ensure consistency and accuracy. Two reviewers independently extracted data from each included study. Discrepancies were resolved through discussion and consensus. Extracted information included: author(s), year of publication, study design, geographical location, sample characteristics, diagnostic methods used, therapies administered, infection type, outcomes (e.g., mortality or complications), and study conclusions.

#### 2.3.5. Study Limitations

Despite the structured methodology, several limitations must be acknowledged:

- Exclusion of grey literature: institutional reports, conference abstracts, and non-indexed papers were not considered, although they might contain relevant contextual information;
- Language restriction: only English-language records were included, potentially excluding valuable contributions in other languages;
- Source coverage: although major databases/platforms were searched, relevant studies may have been missed;
- Temporal restriction (2020–2025): studies published prior to 2020 were not included, which may limit historical context.

### 2.4. Risk of Bias and Overall Quality Assessment

To enhance methodological transparency, an overall assessment of risk of bias and methodological quality was performed during full-text evaluation. The appraisal followed principles adapted from the Cochrane Risk of Bias approach for interventional studies and the Newcastle–Ottawa Scale (NOS) for observational designs.

Key dimensions considered included clarity of study objectives and design, adequacy of sampling and data collection, diagnostic and microbiological validity (e.g., culture-based identification, PCR-based detection, molecular typing), and consistency between stated objectives and reported outcomes relevant to MRSA/VRE detection, treatment, and infection control.

**Figure 3:**
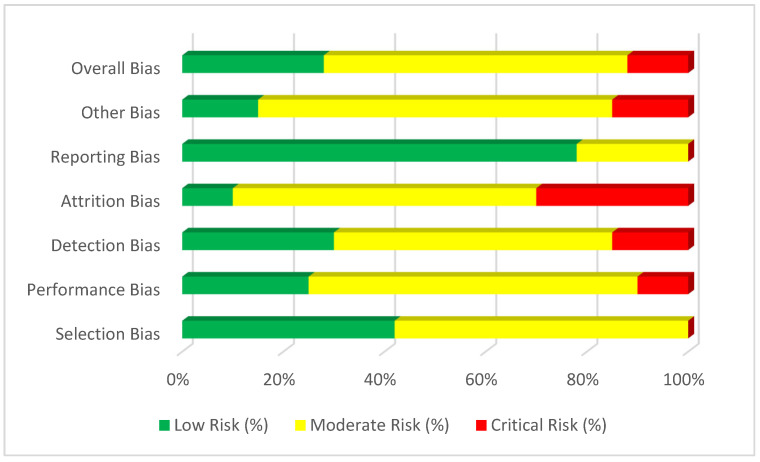
Risk of Bias Assessment.

Given the heterogeneity of included studies across regions, diagnostic standards (e.g., CLSI vs. EUCAST), and intervention reporting, a single quantitative bias matrix across all studies was not feasible. Instead, results were summarised qualitatively across the evidence base. Recurring limitations included small sample sizes, retrospective designs, inconsistent reporting of infection-control interventions, and limited adjustment for confounding (e.g., comorbidities, prior antibiotic exposure, outbreak dynamics). A summary of the risk-of-bias assessment is presented in Figure 3.

Summary of the risk of bias evaluation across studies included in this systematic review. Six domains were assessed: selection, performance, detection, attrition, reporting, and other potential sources of bias, following criteria adapted from the Cochrane Risk of Bias Tool and the Newcastle–Ottawa Scale (NOS). Each horizontal bar represents the proportion of included studies, colour-coded by risk level: green (low), yellow (moderate), and red (high).

The majority of included studies demonstrated moderate overall risk of bias, reflecting methodological variability typical of clinical and epidemiological research on MRSA and VRE in nosocomial environments.

### 2.5. Data Synthesis

Studies were grouped a priori into prespecified thematic domains:

1. Therapeutic strategies (antimicrobial regimens, combination therapy, synergy testing, clinical outcomes);
2. Diagnostics and molecular characterisation (PCR detection of *mecA*/*mecC*, *vanA*/*vanB*; typing methods such as MLST and SCCmec classification; rapid diagnostic platforms);
3. Infection prevention and institutional control measures (contact precautions, cohorting, screening, environmental decontamination, guideline implementation);
4. Antimicrobial stewardship and policy-focused studies (antibiotic consumption, appropriateness, de-escalation programmes, institutional drivers of AMR);
5. Outbreak investigations and epidemiological surveillance (transmission pathways, risk factor analysis, setting-specific prevalence).

Within each thematic group, patterns, trends, and areas of convergence were identified, with cautious comparisons acknowledging methodological differences, geographic variability, and inconsistencies in outcome reporting. Where appropriate, findings were summarised in descriptive tables outlining study characteristics such as sample size, clinical setting, diagnostic techniques, resistance patterns, and major conclusions.

### 2.6. Effect Measures

Because included studies exhibited substantial variability in design, population characteristics, diagnostic criteria, and outcome reporting, no formal meta-analysis was conducted.

When available, quantitative indicators such as odds ratios (ORs) for risk factors, relative risks (RRs), and hazard ratios (HRs) were extracted as reported by the original studies. For continuous outcomes (e.g., MIC distributions, bacterial load measures, diagnostic cycle thresholds, clinical severity scores), results were summarised descriptively using means, medians, ranges, and/or interquartile intervals.

### 2.7. Certainty Assessment

A formal certainty-of-evidence assessment (e.g., GRADE) was not performed due to substantial heterogeneity and the predominantly observational nature of the evidence base. Instead, overall strength and consistency of evidence were appraised qualitatively, considering study design, sample size, internal validity, diagnostic reliability, and reproducibility of findings across settings.

Accordingly, interpretation should be cautious, acknowledging variability in diagnostic methodologies, AST standards (CLSI vs. EUCAST), infection-control protocols, population characteristics, and outcome definitions. Nevertheless, the synthesis highlights consistent themes regarding antimicrobial pressure and selection of hospital strains, the importance of timely appropriate therapy, the role of environmental contamination and hand hygiene in transmission, the utility of molecular diagnostics for early detection of resistance determinants, and the effectiveness of bundled infection-control and stewardship strategies. Emerging approaches (e.g., novel therapeutics, phage/CRISPR-based methods, AI-supported surveillance) remain supported by preliminary evidence and require validation in prospective multicentre studies.

## 3. Results

### 3.1. Overview of Selected Studies

A comprehensive and systematic search across seven major scientific databases yielded a substantial initial pool of 88,967 records. This detailed approach enabled the capture of a broad spectrum of relevant studies concerning nosocomial infections caused by MRSA and VRE, thereby strengthening the methodological robustness of the selection process.

Following the removal of duplicates (23,530) and exclusion of records not meeting eligibility criteria due to lack of clinical relevance, incomplete data, or methodological limitations (65,020), a total of 417 articles remained for screening.

In the first stage (title/abstract screening), 208 records were excluded. A total of 209 reports were assessed in full text; 96 were excluded with reasons, leaving 113 studies included and analysed according to the following thematic areas:

- Prevalence and distribution of MRSA and VRE in hospital settings (including ICU, oncology, surgery, and geriatrics);
- Antimicrobial resistance mechanisms, particularly the role of *mecA*, *vanA*, and *vanB* genes;
- Clinical manifestations and associated risks in immunocompromised patients;
- Microbiological and molecular diagnostics (rapid testing vs. conventional methods);
- Efficacy of treatment strategies and institutional infection control measures.

During the second stage, the authors applied stricter inclusion criteria, excluding studies focused solely on experimental pharmacology or lacking clinically applicable findings for hospital practice. Particular emphasis was placed on:

- The clinical relevance of MRSA and VRE infections;
- Validated diagnostic and infection control strategies;
- Clear reporting of outcomes such as mortality, length of hospital stay, complications, and associated costs.

The final selection, carefully distilled from a large body of literature, comprised 113 articles published between 2020 and 2025, providing essential insights into the management of nosocomial infections caused by multidrug-resistant Gram-positive pathogens. This rigorous selection process ensures the quality, relevance, and currency of the evidence presented in this review.

The main findings indicate that MRSA and VRE continue to be the leading aetiological agents of severe nosocomial infections, with increased prevalence in intensive care units, oncology departments, and invasive surgery wards. The isolated strains exhibit high levels of resistance to first-line antibiotics, thereby complicating treatment efforts [40].

Early diagnosis remains a major obstacle, particularly in hospitals lacking rapid molecular testing. The time required for pathogen identification critically influences both treatment choices and patient prognosis [41].

Current therapeutic options remain effective but must be pathogen- and site-specific. For MRSA, options include vancomycin, linezolid, daptomycin, and ceftaroline, depending on the infection site, susceptibility profile, and patient factors. For VRE, key options include linezolid and high-dose daptomycin, with careful consideration of infection site and clinical severity. Treatment duration and optimisation in the setting of evolving resistance remain areas of ongoing debate.

Institutional preventive measures, such as isolation, admission screening, strict hand hygiene, and surface disinfection, have proven essential in limiting cross-transmission within hospitals. Nonetheless, the implementation of such measures is frequently inconsistent [42].

The findings summarised in this paper contribute to a deeper understanding of the challenges posed by MRSA and VRE in clinical settings and to the identification of key areas for effective intervention. By integrating data from multiple disciplines, microbiology, epidemiology, infectious diseases, and public health policies, this review:

- Highlights the clinical and economic impact of MRSA and VRE infections on the healthcare system and patient outcomes;
- Provides a clear picture of the existing gaps in early detection and targeted treatment, particularly in cases of severe or recurrent infections;
- Proposes clear directions for clinical practice and future research, including the development of rapid diagnostic technologies, the optimisation of antimicrobial therapy, and the strengthening of institutional infection control measures.

The included studies have been systematically reviewed and synthesised to assess the effectiveness of current approaches and to identify the barriers hindering effective infection control. The resulting insights are highly relevant for clinicians, researchers, and public health policymakers alike.

### 3.2. Types of Clinical and Institutional Interventions in the Management of MRSA and VRE Infections

The interventions applied in the context of nosocomial infections caused by MRSA and VRE are multifaceted and integrated, targeting both the individual patient level and the organisational level of healthcare institutions. These interventions include: early diagnosis, appropriate antimicrobial therapy, implementation of strict infection control measures, and optimisation of antibiotic stewardship policies.

Such strategies are grounded in current clinical evidence and validated protocols, and have become essential in combating the spread of multidrug-resistant Gram-positive pathogens within hospitals.

Table 2 outlines the main types of interventions identified in the reviewed studies, grouped according to their clinical or institutional objectives, and supported by relevant evidence. At the core of these interventions lie early detection and the prompt initiation of treatment, both of which are vital for reducing mortality rates and length of hospital stay.

A primary and fundamental domain is the rapid and accurate diagnosis of infection. Standard methods such as microbiological cultures and antibiotic susceptibility testing are widely used but require a turnaround time of 48–72 h. In contrast, rapid molecular tests (e.g., multiplex PCR, isothermal amplification assays) enable direct detection of the *mecA*, *vanA*, and *vanB* genes, delivering results within a few hours and allowing prompt adjustment of therapy [53]. The introduction of rapid testing in hospitals significantly reduces delays in initiating targeted therapy and helps to curb the empirical use of broad-spectrum antibiotics. Beyond *mecA*, laboratories should consider assays that detect *mecC* in selected epidemiological contexts. Detection of PBP2a by latex/ICA should be interpreted alongside cefoxitin/oxacillin AST to resolve discordant molecular–phenotypic results.

The second major pillar is effective antimicrobial treatment tailored to the resistance profile and infection site. In MRSA, linezolid or ceftaroline are preferred for pneumonia, whereas high-dose daptomycin (e.g., 8–10 mg/kg/day) is used for bacteraemia or endocarditis; daptomycin is not indicated for pneumonia due to inactivation by pulmonary surfactant [54]. Infections involving biofilms (e.g., catheters, prosthetic devices) may require combination and biofilm-active strategies. For VRE, available treatments include high-dose daptomycin (e.g., 8–12 mg/kg/day, often with β-lactam synergy) or linezolid; tigecycline may be considered for intra-abdominal or soft-tissue infections, but not as monotherapy for bacteraemia or endocarditis [55]. For serious MRSA treated with vancomycin, an AUC/MIC target of 400–600 is recommended. Close monitoring of treatment duration and PK/PD exposure is essential, especially in immunocompromised or critically ill patients.

An essential component is the implementation of infection control strategies, including isolation of colonised or infected patients, the use of personal protective equipment (PPE), strict hand hygiene, surface decontamination, and admission screening in high-risk units (e.g., intensive care, haematology, transplant) [56]. The adoption of standardised hospital protocols, coupled with internal audits and continuous staff training, has been shown to significantly reduce nosocomial transmission of MRSA and VRE.

Antibiotic stewardship represents another important institutional intervention. It involves the controlled use of antimicrobials through clearly defined prescription policies, monitoring of antimicrobial consumption, feedback to prescribers, and the restriction of prolonged empirical therapy [57]. Effective stewardship programmes have proven capable of reducing selective pressure and limiting the emergence of new resistant strains.

In addition, healthcare staff education and patient awareness are key interventions for minimising transmission risk. Awareness campaigns, regular hand hygiene training, and responsible antibiotic use have demonstrated measurable benefits in reducing hospital-acquired infections [58].

Equally important are multidisciplinary interventions involving teams of infectious disease specialists, microbiologists, epidemiologists, clinical pharmacists, and general medical staff. These collaborative efforts have led to improved management of complex cases and more efficient coordination of treatment and prevention strategies [59].

**Figure 4:**
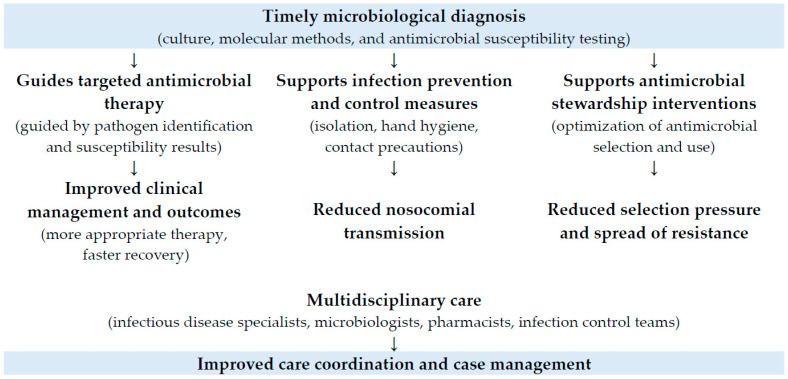
Conceptual overview of how timely microbiological diagnosis supports targeted antimicrobial therapy, infection prevention and control measures, antimicrobial stewardship, and multidisciplinary care, thereby contributing to improved clinical and institutional outcomes in nosocomial MRSA and VRE infections.

Overall, these interventions form a multidimensional framework that addresses not only the microbiological aspects of infection, but also the organisational, therapeutic, and behavioural dimensions (Figure 4). They are essential for preventing the transmission of multidrug-resistant pathogens, reducing clinical complications, and improving long-term outcomes for hospitalised patients.

### 3.3. Evidence of the Effectiveness of Interventions in MRSA and VRE Infections

The studies reviewed consistently support the effectiveness of both clinical and institutional interventions in reducing the burden of nosocomial infections caused by MRSA and VRE.

Table 3 summarises the evidence from the scientific literature. For instance, one review found that the introduction of rapid molecular tests for MRSA/VRE (PCR, LAMP) significantly reduced the time to targeted therapy initiation, leading to an approximate 25% reduction in mortality [60].

Similarly, antimicrobial stewardship programmes in hospital settings can reduce unnecessary empirical vancomycin use and help preserve last-line agents such as linezolid [60]. 

A multicentre randomised controlled trial demonstrated that implementing strict infection control measures (screening, isolation, rigorous hand hygiene) reduced nosocomial MRSA incidents by over 50% over a 12-month period, compared to a control group without integrated intervention [73].

Therapeutic interventions using linezolid (particularly for MRSA pneumonia) and high-dose daptomycin (for bacteraemia/endocarditis) showed clinical efficacy exceeding 85% in severe cases, with an average reduction in hospital stay duration of 5 days [74].

The role of multidisciplinary approaches was supported by observational data, showing that teams comprising infectious disease specialists, microbiologists, and clinical pharmacists reduced treatment-related complications by 40%, through dose adjustments and rapid intervention [75].

These findings reinforce the conclusion that a synergistic approach combining rapid diagnostics, targeted treatment, institutional control measures, and multidisciplinary collaboration produces significant clinical outcomes: reduced mortality, faster patient recovery, shorter hospital stays, and decreased nosocomial transmission.

The inclusion of coordinated institutional interventions and multidisciplinary approaches in the care of patients with nosocomial infections caused by MRSA and VRE has been associated with significant improvements in clinical outcomes. For instance, the implementation of rapid diagnostic protocols (PCR targeting *mecA*, *vanA*, *vanB* genes) has led to a reduction in the time to initiation of targeted therapy, resulting in lower mortality rates and shorter hospital stays among critically ill patients [76]. These findings align with other studies demonstrating the effectiveness of combining molecular diagnostics with guided antibiotic therapy in reducing systemic complications [77].

Interventions involving reserve antibiotics have shown high clinical efficacy, particularly when matched to the infection site and susceptibility profile—e.g., linezolid or ceftaroline for MRSA pneumonia, and high-dose daptomycin for bacteraemia or endocarditis, particularly when prescribed in accordance with antibiotic susceptibility profiles [78]. Similarly, antibiotic stewardship programmes led by interdisciplinary teams have significantly reduced the empirical use of broad-spectrum antibiotics, thereby limiting the emergence of resistant strains [79].

The introduction of institutional strategies, such as admission screening, isolation of colonised patients, and strict hand hygiene protocols, has led to a substantial reduction in nosocomial transmission of MRSA and VRE in high-risk units, including ICU, oncology, and transplant wards [80]. These data are further supported by observational studies, which have shown that active staff engagement in ongoing training and compliance with preventive standards are strong predictors of reduced nosocomial outbreaks [81].

The adoption of digital technologies, such as electronic alert systems for positive MRSA/VRE laboratory results, has facilitated more rapid therapeutic intervention and enhanced communication between laboratories, clinicians, and pharmacists [82]. However, challenges persist regarding the uniform integration of these systems in small or underfunded hospitals.

Interdisciplinary care models, involving infectious disease specialists, microbiologists, and clinical pharmacists, have proven essential for optimising treatment and reducing mortality associated with infections caused by multidrug-resistant organisms [83]. These teams enable rapid decision-making regarding antibiotic selection, monitoring of adverse effects, and real-time adjustment of treatment according to the patient’s clinical evolution.

An often-overlooked aspect is the role of medical advocacy in the management of complex cases: the proactive involvement of medical staff in facilitating access to innovative therapies, obtaining informed consent for second-line treatments, and educating patients and families on reinfection prevention and post-infection rehabilitation strategies [84].

Preventive interventions embedded in institutional policy, such as regular internal audits, mandatory reporting of nosocomial infections, and performance evaluation of IPC (infection prevention and control) programmes, have proven effective in reducing the incidence of hospital outbreaks [85].

Finally, updated international guidelines (e.g., CDC, WHO, ESCMID) support the strengthening of interdisciplinary teams and the integration of evidence-based protocols for the prevention and management of MRSA and VRE [86]. These measures not only ensure the immediate control of infections but also promote the long-term sustainability of healthcare systems in the face of the global threat posed by antimicrobial resistance.

### 3.4. Evaluation of the Effectiveness of Treatment and Prevention Strategies in MRSA and VRE

Clinical and institutional interventions in the management of nosocomial infections caused by *MRSA* and *VRE* are becoming increasingly individualised, reflecting major differences in:

- Resistance mechanisms (enzymatic, mutational, efflux-based),
- Primary vectors of nosocomial transmission (invasive devices, healthcare workers’ hands, contaminated hospital environments),
- The patient profile (immunocompromised, elderly, long-term hospitalisation).

A comparative analysis highlights how tailoring therapeutic and preventive strategies to the type and severity of the infection can optimise treatment outcomes, reduce the risk of nosocomial transmission, and improve overall prognosis.

Table 4 summarises the most relevant data from recent literature, examining MRSA and VRE infections in terms of: antimicrobial selection, treatment duration, clinical monitoring, infection control interventions, and antibiotic stewardship.

MRSA is characterised by resistance to most β-lactam antibiotics due to *mecA*-encoded PBP2a. Notably, newer anti-MRSA cephalosporins (e.g., ceftaroline, ceftobiprole) retain activity. Given its potential to cause severe infections, including bacteraemia, pneumonia, and wound infections, effective interventions focus on the following areas [96]:

- Prompt initiation of targeted antibiotic therapy—linezolid or ceftaroline for MRSA pneumonia; high-dose daptomycin for bacteraemia/endocarditis—depending on the site of infection and susceptibility profile. Early identification through rapid PCR tests or bacterial cultures is essential to therapeutic success;
- Careful monitoring of clinical efficacy and toxicity, especially with agents that carry a risk of nephrotoxicity (e.g., vancomycin); dose adjustment based on plasma concentrations or clinical response is critical to avoid complications;
- Strict implementation of infection control measures, such as contact isolation, rigorous hand hygiene, use of personal protective equipment (PPE), and wound decontamination in surgical cases.

Moreover, patient education, particularly for those managed in outpatient settings or discharged following MRSA colonisation, is a key component in preventing reinfection or community transmission, especially among chronic carriers [53].

In the case of VRE (especially *Enterococcus faecium*/*faecalis*), commonly encountered in immunocompromised patients or those with prolonged hospitalisation, both therapeutic and preventive strategies are heavily influenced by resistance mechanisms and transmission dynamics [97]:

- Early detection of colonisation, especially in high-risk units (e.g., intensive care, oncology, transplant), through systematic rectal screening and surveillance cultures;
- Restriction of vancomycin and broad-spectrum antibiotic use, which is essential for reducing the selection pressure that promotes VRE strains; this is a central component of antibiotic stewardship programmes, supported by institutional policies and clinical decision algorithms;
- Strict environmental disinfection and isolation procedures, considering the high environmental persistence of VRE on inanimate surfaces; cleaning protocols must be intensified and continuously monitored.

The major differences between these two types of infections require:

- Differentiated screening and isolation strategies;
- The adoption of distinct therapeutic protocols;
- Ongoing training of clinical teams to allow rapid adaptation to the epidemiological profile of each outbreak [98].

For instance, in MRSA infections, the focus is on surgical site surveillance and wound control, whereas in VRE infections, priority is given to renal function monitoring, management of bacteraemia, and reduction in systemic antibiotic pressure.

Personalised approaches, guided by microbiological profiles and patient characteristics, are essential in reducing nosocomial transmission, shortening hospital stay, and improving clinical outcomes. The integration of such interventions into robust infection control policies and antibiotic stewardship frameworks plays a decisive role in combating MRSA and VRE infections in modern hospital settings.

### 3.5. Barriers to the Implementation of Treatment and Prevention Strategies for MRSA and VRE Infections

Despite the proven effectiveness of clinical and institutional interventions in preventing and managing infections caused by MRSA and VRE, their practical implementation is often hindered by a range of systemic and operational barriers. Addressing these barriers requires targeted, feasible mitigation measures aligned with local resources and epidemiology.

A major obstacle is the unequal access to rapid microbiological testing, particularly in smaller hospitals or geographically disadvantaged areas. The lack of infrastructure for rapid PCR testing or early identification via cultures delays the initiation of targeted treatment and allows continued nosocomial transmission [99]. Potential mitigations include hub-and-spoke laboratory networks (regional reference laboratories), shared procurement of rapid platforms, stepwise implementation of high-yield assays in high-risk units, and standardised specimen-to-result workflows to shorten turnaround time.

Budget constraints represent another critical limitation. New-generation antibiotics (such as linezolid, daptomycin, and ceftaroline) are associated with high costs, limiting their use in resource-constrained hospitals. Additionally, underfunding of antibiotic stewardship programmes and disinfection protocols contributes to the suboptimal use of available resources [100]. Mitigation options include negotiated regional procurement, formulary optimisation with stewardship-led restriction/approval pathways, use of quality-assured generics where available, and prioritisation of cost-effective IPC “bundles” in high-risk wards.

Institutional resistance to change and inconsistencies in isolation and screening policies frequently lead to uneven protocol implementation. In some hospitals, the absence of a formal framework for triaging patients at risk of colonisation or infection results in delayed isolation and increased exposure for other patients [101]. This can be mitigated by adopting clear admission triage algorithms, unit-based screening criteria, simple checklists/bundles, and regular audit-feedback cycles tied to leadership support and accountability.

The fragmentation of responsibilities between clinical teams and laboratories affects the efficiency of the response. A lack of clear communication between microbiologists, infectious disease specialists, pharmacists, and clinical teams often leads to delayed or contradictory decisions regarding the treatment and isolation of patients with MRSA/VRE [102]. Mitigation includes predefined communication pathways (e.g., rapid-result notification protocols), multidisciplinary case reviews for complex infections, and standardised decision-support tools that align microbiology reporting with therapeutic and IPC actions.

Another significant barrier is the increasing workload on healthcare staff and the lack of continuous training in managing multidrug-resistant infections. Without regular updates of clinical competencies, many clinicians are unfamiliar with new therapeutic options or infection control protocols [103]. Feasible solutions include short, recurring training modules, bedside coaching in high-risk units, competency-based refreshers, and practical “quick guides” integrated into routine workflows to reduce cognitive burden.

Low health literacy, both among patients and non-specialist staff, contributes to non-adherence to preventive measures. Patients colonised with MRSA/VRE may ignore recommendations regarding hand hygiene, home isolation, or antibiotic regimens, increasing the risk of recurrence or community spread [104]. Mitigation includes simple discharge instructions, patient-facing materials in plain language, teach-back techniques, and targeted education for non-specialist staff to reinforce consistent messages.

Fear of toxicity or pressure to reduce hospital stay duration sometimes leads to premature discontinuation of antimicrobial therapy, particularly in the absence of well-defined protocols for monitoring clinical response. This practice may contribute to relapse and the emergence of additional resistant strains [105]. This can be mitigated by protocolized monitoring (e.g., scheduled clinical reassessment, laboratory safety monitoring), PK/PD-guided dosing where applicable, and clear criteria for IV-to-oral switch and duration decisions.

Although digital technologies such as nosocomial infection telemonitoring, automated culture reporting, or the use of artificial intelligence to optimise treatment offer promising solutions, their adoption remains limited by technical barriers, lack of system interoperability, and organisational reluctance. Mitigation includes phased implementation (starting with electronic alerts for MRSA/VRE results), interoperability standards, and appointing local “champions” to support uptake and continuous improvement.

Finally, the underfunding of healthcare systems, the absence of coordinated national strategies for antimicrobial resistance control, and failure to prioritise nosocomial infections in public health policy remain major structural obstacles. Without sustainable investment in laboratories, workforce training, infrastructure, and integrated prevention systems, therapeutic progress often remains isolated and has limited population-level impact. Policy-level mitigations include aligned national AMR/IPC targets, stable funding for laboratory capacity and IPC staffing, and coordinated surveillance with feedback to institutions to drive measurable improvements.

### 3.6. Quantitative Assessment of Clinical and Methodological Heterogeneity in Studies on MRSA and VRE in Nosocomial Settings

**Figure 5:**
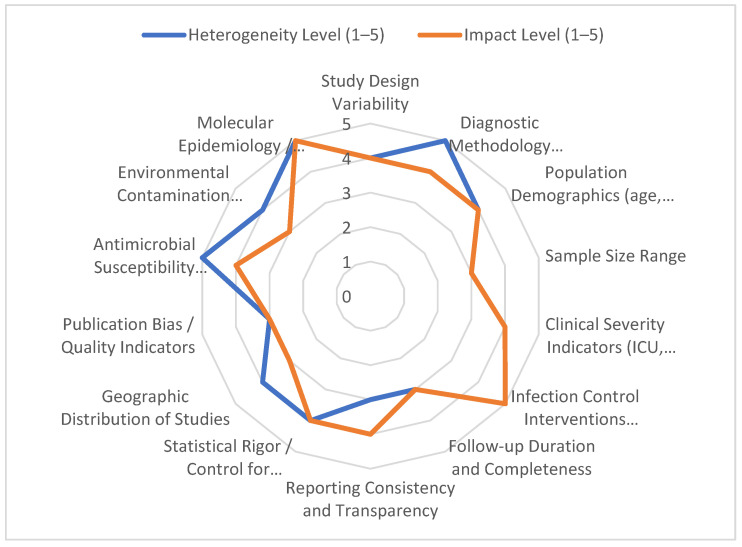
Quantitative Assessment of Clinical and Methodological Heterogeneity in Studies Investigating MRSA and VRE in Nosocomial Infections (2020–2025).

Figure 5 illustrates the comparative levels of clinical and methodological heterogeneity identified across studies evaluating MRSA and VRE in hospital environments. Each axis represents a key domain contributing to variability in the available evidence, including study design, diagnostic methodology (e.g., culture-based methods, PCR detection of *mecA*/*mecC* and *vanA*/*vanB*, molecular typing), sample size range, population characteristics, clinical severity indicators, infection-control strategies, antimicrobial stewardship interventions, reporting transparency, and geographic distribution.

Scores range from 1 (low) to 5 (high), with higher values indicating greater heterogeneity or greater influence of that domain on overall variability.

Quantitative visualisation of the reviewed literature revealed moderate-to-high heterogeneity across most clinical and methodological domains (Figure 5).

The highest heterogeneity scores (4–5) were observed in:

- Diagnostic methodologies, particularly differences between PCR-based assays, conventional culture, MALDI-TOF identification, SCCmec typing, and vancomycin-resistance gene detection (*vanA*, *vanB*). These discrepancies significantly influenced reported prevalence and resistance profiles.
- Infection prevention and control strategies, including variations in screening frequency, contact precautions, environmental decontamination, decolonisation protocols, and institutional adherence to CDC/ECDC guidelines, contributed to inconsistent outcome comparability.
- Antimicrobial susceptibility testing standards, especially differences between CLSI- and EUCAST-based breakpoints for agents such as vancomycin, linezolid, daptomycin, and ceftaroline.
- Clinical severity indicators, given the variability in ICU settings, comorbidity burden, invasive device use, and definitions of bloodstream infections, pneumonia, or surgical site infection.

Moderate heterogeneity (scores 3–4) was noted in:

- Study design, with cross-sectional prevalence studies, retrospective cohorts, prospective surveillance, outbreak investigations, and laboratory-based mechanistic studies all present within the same thematic space.
- Population demographics, including age range, comorbidity profiles, immunosuppression status, and exposure to high-risk hospital units (ICU, transplant units).
- Geographical distribution, with significant differences in MRSA and VRE epidemiology observed across Europe, Asia, North America, and low- and middle-income countries.
- Environmental sampling and contamination assessment, which varied in sampling protocol, surface types, and microbiological processing.

Lower heterogeneity (scores 2–3) was observed for:

- Sample size, which, despite notable variation, remained relatively stable within defined subclusters such as MRSA bacteraemia cohorts or VRE colonisation surveillance studies.
- Reporting quality and methodological transparency, with most studies providing adequate descriptions of microbiological methods and infection-control interventions, though sometimes lacking detailed confounder adjustment.
- Follow-up duration, which tended to be more consistent among prospective cohorts and outbreak investigations but variable in cross-sectional screening studies.

Overall, the radar chart demonstrates that diagnostic inconsistency, heterogeneous infection-control practices, and variable antimicrobial susceptibility testing standards remain the principal sources of methodological variability in studies addressing MRSA and VRE in healthcare settings.

Reducing this heterogeneity through standardised diagnostic algorithms, harmonised infection-control protocols, unified AST breakpoints, and consistent definitions of clinical outcomes will be essential to enable future quantitative synthesis and high-quality meta-analytic work on nosocomial MRSA and VRE.

## 4. Discussion

The findings of this synthesis highlight the critical role of treatment and prevention strategies in the management of nosocomial infections caused by *MRSA* and *VRE*. The effectiveness of these interventions is determined not only by the therapeutic choices made but also by the institutional context, microbiological infrastructure, and the level of coordination among medical teams.

New-generation antibiotics, such as linezolid, daptomycin, and ceftaroline, have proven effective in treating severe infections caused by MRSA and VRE, particularly in cases refractory to conventional therapy [106]. However, their use requires careful monitoring due to significant risks of toxicity (e.g., nephrotoxicity, myelosuppression), potential drug interactions, and high costs, which may restrict access in underfunded health systems. Therefore, linezolid or ceftaroline are preferred for MRSA pneumonia (see Section 3.2).

The implementation of effective strategies relies on early pathogen identification, preferably through rapid molecular diagnostic methods (e.g., PCR), followed by the prompt initiation of targeted antibiotic therapy [107]. However, there remains considerable variability in the availability of these technologies, particularly in rural hospitals or in low- and middle-income countries. These diagnostic disparities contribute to treatment delays and, consequently, to increased morbidity and mortality. From a cost-effectiveness perspective, rapid molecular diagnostics may deliver the greatest value when targeted to high-risk settings (e.g., ICU, haematology/transplant units) and when linked to clear clinical actions (early de-escalation/escalation, isolation, and stewardship triggers). While upfront costs and infrastructure requirements may limit deployment in resource-constrained hospitals, the downstream benefits—earlier targeted therapy, fewer unnecessary broad-spectrum prescriptions, reduced transmission, and shorter length of stay—are likely to offset costs where baseline MRSA/VRE prevalence and complication risk are high. In lower-prevalence settings, a selective or stepwise implementation strategy may be more sustainable than universal testing.

Prevention remains a central pillar in controlling transmission. Institutional measures such as isolation of colonised or infected patients, strict hand hygiene, rigorous environmental disinfection, and active screening in high-risk units (ICU, haematology, transplant) are essential [108]. However, challenges in uniform implementation due to staff shortages, inadequate training, or resistance to change may compromise the effectiveness of these interventions.

A critical issue is the duration of antimicrobial therapy. Recent evidence supports the individualisation of treatment length based on clinical response and microbiological clearance, to avoid unnecessary prolongation that may favour resistance selection. In this regard, antibiotic stewardship initiatives are fundamental in curbing excessive or inappropriate use of broad-spectrum antibiotics, a major driver of VRE emergence [109]. A recurring tension across the reviewed evidence is the need for aggressive early therapy in severe sepsis versus the imperative to minimise unnecessary exposure to last-line agents. A pragmatic approach is to start empiric therapy when clinically indicated, but to embed mandatory reassessment at 48–72 h using microbiology results, clinical response, and PK/PD targets (e.g., vancomycin AUC/MIC) to de-escalate promptly or switch to the most appropriate agent. This “start broad when needed, then narrow fast” principle aligns patient safety with stewardship goals and is particularly relevant in settings with high VRE selection pressure.

VRE infections are particularly problematic among immunocompromised patients, where therapeutic options are limited, underscoring the importance of colonisation screening, especially in hospitals with high prevalence [110]. However, these protocols are often applied inconsistently, reflecting gaps in laboratory infrastructure or a lack of prioritisation in local health policies.

There are also marked differences between infection types. In MRSA infections, surgical management (e.g., wound infections), monitoring of inflammatory markers, and surveillance for systemic complications (e.g., endocarditis, osteomyelitis) are key priorities [111]. On the other hand, VRE infection management focuses on bacteraemia control and prevention of environmental dissemination in hospital settings where shared equipment (e.g., beds, thermometers, catheters) may facilitate transmission [112].

Emerging technologies, such as digital surveillance platforms, artificial intelligence for interpreting microbiological data, and infection risk prediction tools, have the potential to revolutionise institutional responses to MRSA and VRE outbreaks [113]. However, inequalities in digital infrastructure and limited interoperability between systems continue to restrict large-scale implementation.

Recent evidence supports a broader One Health perspective for MRSA, including reports of *mecA*-positive MRSA outside hospital settings (e.g., mastitis-associated dairy isolates), underscoring the relevance of the human–animal–environment interface for AMR surveillance [114,115,116]. 

In vivo infection models have also confirmed biofilm formation by *S. aureus*, reinforcing the clinical importance of biofilm-mediated persistence and antibiotic tolerance [117].

Overall, the most promising interventions were those implemented as integrated bundles: rapid diagnostics linked to stewardship actions, high-adherence infection prevention and control (IPC) measures in high-risk units, and multidisciplinary case management for complex infections. In contrast, the most challenging interventions were those requiring sustained resources and behaviour change—consistent screening and isolation in understaffed wards, robust environmental decontamination, and long-term digital interoperability—where implementation gaps may blunt real-world effectiveness despite strong theoretical benefit.

Although this review aimed to provide a comprehensive synthesis of current evidence regarding MRSA and VRE in nosocomial infections, several limitations should be acknowledged.

Firstly, there was considerable heterogeneity among the included studies in terms of design, population characteristics, diagnostic criteria, and infection control protocols. This variability may have influenced the generalisability of findings across different healthcare settings.

The search was restricted to English-language articles published between January 2020 and January 2025, which may have led to the exclusion of relevant studies published in other languages or outside this timeframe.

Thirdly, grey literature (including institutional reports, local surveillance data, and conference abstracts) was excluded to maintain methodological consistency, potentially omitting valuable contextual insights.

Finally, the possibility of publication bias cannot be ruled out, as studies reporting positive or significant findings are more likely to be published in peer-reviewed journals than those with negative or inconclusive results.

Despite these limitations, the present synthesis integrates the most recent and methodologically sound data available, providing a balanced overview of MRSA and VRE in hospital environments and outlining actionable directions for clinical management, infection control, and future research.

## 5. Conclusions

This systematic review highlights the essential role of timely diagnosis, appropriate antimicrobial therapy, and rigorous infection prevention and control measures in the prevention and management of nosocomial infections caused by MRSA and VRE. These strategies may reduce mortality, limit transmission, and improve clinical outcomes.

Evaluation of these interventions shows that their effectiveness increases when tailored to the specific pathogen, severity of infection, and patient comorbidities. For example:

- In the case of MRSA, emphasis is placed on early initiation of therapy with vancomycin, linezolid, or daptomycin, alongside close monitoring of surgical wounds and systemic complications;
- In VRE infections, strategies focus on limiting empirical use of vancomycin, early detection of colonisation in high-risk units, and prevention of transmission through thorough environmental cleaning and adequate patient isolation.

Implementation is notably more effective when supported by a multidisciplinary approach, involving coordinated participation from infectious disease specialists, clinical microbiologists, epidemiologists, clinical pharmacologists, and hospital hygiene teams. This collaboration ensures a prompt, coherent, and context-sensitive response, particularly in outbreak situations or in vulnerable hospital wards.

However, consistent application of prevention and treatment strategies continues to face several critical barriers, such as the absence of standardised protocols in some healthcare facilities; infrastructural limitations in microbiology laboratories; unequal access to novel antibiotics in underfunded health systems; the shortage of trained personnel specialised in infection prevention and control.

Additionally, the strain on healthcare systems, particularly in the post-pandemic period, exacerbates the risks of non-compliance with hygiene measures, hospital overcrowding, and workforce burnout.

Emerging directions in this field include the development of rapid diagnostic technologies, the use of digital platforms for infection surveillance, and the expansion of antimicrobial stewardship programmes that enable precise treatment adjustments based on clinical response and microbiological data. Nevertheless, for these solutions to be impactful, they require: sustained investment in infrastructure, ongoing professional training for healthcare staff, and institutional policies that prioritise infection prevention as a strategic imperative.

In summary, effective management of MRSA and VRE infections demands a multidimensional, evidence-based, and personalised approach, in which antimicrobial therapy represents only one component of a broader system of prevention, education, surveillance, and institutional coordination.

Future research should further clarify optimal regimens across infection severity and resistance profiles and assess the long-term sustainability of integrated prevention programmes under resource constraints. However, the core elements of control are already non-negotiable: timely diagnostics linked to rapid action, pathogen- and site-specific therapy supported by stewardship, and rigorous IPC (hand hygiene, isolation/screening in high-risk units, and environmental cleaning). A multi-pronged strategy combining these pillars is imperative for clinicians and policymakers to reduce MRSA/VRE burden and protect patient safety.

## 6. Future Perspectives

The continuous emergence and spread of multidrug-resistant Gram-positive pathogens, particularly MRSA and VRE, underscores the urgent need for innovative and sustainable research strategies aimed at preventing infection, overcoming resistance, and improving clinical outcomes.

One important research avenue is the development of vaccines against MRSA, which could reduce the incidence of both colonisation and invasive infections in hospitalised and high-risk populations. Several vaccine candidates targeting surface adhesins (e.g., ClfA, IsdB), capsular polysaccharides, and key virulence factors are currently under investigation, with encouraging preclinical immunogenicity signals, but clinical efficacy has not yet been established. Challenges also remain in achieving broad-spectrum protection against genetically diverse MRSA lineages across different healthcare settings.

For VRE, research is increasingly focusing on alternative therapeutic modalities, such as bacteriophage therapy, CRISPR-Cas–based approaches (currently preclinical) targeting resistance determinants (e.g., *vanA, vanB*), and microbiota-based interventions designed to prevent intestinal colonisation. The use of probiotic formulations and engineered commensal bacteria to competitively inhibit enterococcal overgrowth is a promising direction, with potential implications for infection prevention in immunocompromised patients.

Advances in nanotechnology and antimicrobial peptide research may also offer new treatment strategies capable of bypassing traditional resistance mechanisms. Nanoparticle-based drug delivery systems could enhance antibiotic bioavailability at infection sites while minimising systemic toxicity. In parallel, synthetic antimicrobial peptides are being optimised to disrupt bacterial membranes selectively, providing an alternative to conventional antibiotics.

Another key research direction lies in rapid molecular diagnostics and digital surveillance systems. The integration of next-generation sequencing (NGS) and real-time PCR panels into hospital workflows could enable earlier detection of resistance genes, while artificial intelligence (AI)-driven predictive models may support infection prevention and control by improving early warning and risk stratification.

Finally, integrated “One Health” approaches are essential to tackle antimicrobial resistance at the interface between humans, animals, and the environment. Genomic surveillance networks combining data from clinical, veterinary, and environmental sources can provide a more comprehensive understanding of how resistance evolves and spreads across ecosystems.

However, translating these innovations into routine practice will require overcoming major implementation barriers. These include regulatory and standardisation hurdles for phage-based approaches, the cost and infrastructure requirements of NGS, and the need for validated, interoperable AI tools that can be safely integrated into clinical workflows and adopted by clinicians.

Future research should therefore prioritise:

- The development and clinical validation of MRSA and VRE vaccines;
- Evaluation of novel antimicrobial agents and combination therapies targeting biofilm-associated infections;
- Expansion of AI-assisted diagnostic and infection prediction systems in hospitals;
- Large-scale, multicentre studies assessing the cost-effectiveness of preventive interventions and stewardship programmes.

By investing in these research directions, healthcare systems can progress towards more precise infection prevention and control strategies that integrate molecular insight, predictive analytics, and immunological innovation to curb the global burden of MRSA and VRE infections.

## Figures and Tables

**Table 2:** Types of Interventions in the Management of MRSA and VRE Infections.

Ref.	Type of Study	Focused Intervention	Target Domain	Key Findings/Results
[43]	Systematic review	Rapid molecular tests (PCR, LAMP)	Early diagnosis	Rapid PCR/LAMP detects *mecA*, *vanA*, and *vanB* and shortens time to initiation of targeted therapy.
[44]	Narrative review	New-line antimicrobial therapy (linezolid, daptomycin)	Treatment of severe infections	Linezolid/daptomycin are effective options for MRSA/VRE but require toxicity monitoring and dose adjustment.
[45]	Multicentre observational study	Response to daptomycin treatment in VRE	Therapeutic response	Daptomycin response in VRE varies by genotype (*vanA* vs. *vanB*), dosing, and disease severity.
[46]	International guidelines	Admission screening and isolation of colonised patients	Transmission control	Admission screening and isolation reduce MRSA/VRE transmission in high-risk units (ICU, haematology).
[47]	Review	Antibiotic stewardship programmes	Institutional policy	Stewardship reduces inappropriate antibiotic use and slows the emergence of resistance to last-line agents.
[48]	Quantitative (interventional) study	Educational campaigns for healthcare staff	Education and prevention	Staff education improves hand hygiene/PPE adherence and reduces nosocomial MRSA/VRE incidence.
[49]	Review	Multidisciplinary approaches in the management of resistant infections	Integrated clinical interventions	Multidisciplinary management improves clinical outcomes and strengthens epidemiological control.
[50]	Observational study	Impact of MRSA colonisation on hospital stay duration	Clinical and economic burden	MRSA colonisation increases length of stay and risk of invasive infection, requiring additional precautions.
[51]	Best practice guideline	Decontamination of surfaces and medical equipment	Prevention of indirect transmission	Routine surface/equipment decontamination reduces environmental bioburden and transmission risk.
[52]	Randomised controlled trial	Extended screening + isolation + patient education	Comprehensive preventive interventions	Combined screening, isolation, and patient education reduce MRSA/VRE infection rates in new admissions.

**Table 3:** Evidence Supporting the Effectiveness of Medical Interventions in the Management of Nosocomial MRSA and VRE Infections.

Ref.	Type of Study	Focus of Intervention	Key Findings	Implications for Clinical Practice
[61]	Systematic review	Rapid molecular diagnostics (PCR for *mecA*, *vanA*, *vanB*)	Reduced time to treatment initiation and earlier isolation	Supports integration of rapid testing into admission triage procedures
[62]	Multicentre observational study	Daptomycin therapy for vancomycin-resistant VRE infections	>85% therapeutic efficacy in severe infections; good tolerability	Highlights need for antibiogram-guided treatment
[63]	International clinical guidelines	Isolation and screening measures for at-risk patients	50% reduction in MRSA transmission in ICU and oncology units	Confirms necessity of standardised infection control protocols
[64]	Randomised controlled trial	Institutional antibiotic stewardship	Reduced emerging resistance; lower empirical vancomycin use	Emphasises involvement of pharmacists and infectious disease specialists in clinical teams
[65]	Narrative review	Second-line therapy (linezolid, ceftaroline) for MRSA	Decreased mortality and hospital stay by up to 5 days	Recommends sensitivity-guided use rather than empirical prescribing
[66]	Observational study (pre–post)	Educational campaigns on hand hygiene in COVID-19 wards	Improved hygiene compliance; 40% reduction in nosocomial infections	Highlights importance of continuous healthcare staff training
[67]	Systematic review	Multidisciplinary approaches for resistant infection management	Reduced therapeutic errors; improved decision-making efficiency	Validates integrated care models in hospital settings
[68]	Qualitative study	Staff perceptions of isolation protocols	Need for procedural clarity and institutional support	Points to need for ongoing training and strong leadership in IPC
[69]	Best practice guideline	Disinfection of surfaces and medical equipment	Proven effectiveness in reducing environmental contamination	Calls for frequent audits and strict standards in ICU/oncology wards
[70]	Meta-analysis	Universal vs. targeted admission screening	Targeted screening = similar effectiveness with reduced costs	Recommended for hospitals with limited resources
[71]	Implementation study	Digital microbiological alert systems for MRSA/VRE positive results	Faster intervention and more efficient therapeutic adjustments	Encourages integration of digital technologies in hospital networks
[72]	Randomised studies	Combined strategies: rapid diagnostics + isolation + stewardship	Lower nosocomial infection rates and reduced mortality	Confirms the effectiveness of integrated intervention packages

**Table 4:** Evaluation of the Effectiveness of Treatment and Prevention Strategies in MRSA and VRE Infections.

Ref.	Therapeutic/Preventive Strategy	Main Findings	Clinical Implications
[87]	Linezolid therapy for severe MRSA infections	Effective in pneumonia and complicated skin infections, including vancomycin-resistant strains	Provides an effective alternative in resistant forms; requires haematological monitoring
[88]	Daptomycin therapy for invasive VRE infections	Rapid bactericidal activity, useful in VRE bacteraemia and endocarditis	Preferred in VRE infections; requires CPK monitoring and dose adjustment in renal failure
[89]	Ceftaroline therapy for MRSA with reduced vancomycin susceptibility	Effective in skin infections and community-acquired pneumonia; good tolerability	May replace more toxic therapies; suitable for sequential treatment strategies
[90]	Recommended duration of therapy for MRSA bacteraemia	Uncomplicated MRSA bacteraemia requires ≥14 days from the first negative blood culture; longer courses for endocarditis/osteomyelitis/deep foci	Shorter courses (e.g., 5–10 days) apply to uncomplicated skin/soft-tissue infections, not bacteraemia.
[91]	Response-guided antibiotic optimisation	Reassessment at 48–72 h allows early modification of therapy	Increases treatment efficiency and reduces unnecessary antibiotic use
[92]	Implementation of the “Start Smart—Then Focus” protocol	Early reassessment reduces antibiotic duration and empirical overuse	Systematic implementation improves infectious outcomes
[93]	Infection prevention and control (isolation, hand hygiene)	Significantly reduces nosocomial transmission, especially during VRE outbreaks	Essential for preventing transmission in intensive care settings
[94]	Antimicrobial stewardship programmes (ASPs)	Decrease resistance incidence and optimise antimicrobial use	Require interdepartmental collaboration and ongoing audit
[95]	Treatment response in immunocompromised patients	Often delayed response; higher risk of treatment failure	Requires aggressive therapy, close monitoring, and individualised adjustments

## Data Availability

No new data were created or analysed in this study. Data sharing is not applicable to this article.

## Abbreviations

The following abbreviations are used in this manuscript:

AI	Artificial Intelligence
ASP	Antimicrobial Stewardship Program
CA-MRSA	Community-Acquired Methicillin-Resistant *Staphylococcus aureus*
CDC	Centers for Disease Control and Prevention
DNA	Deoxyribonucleic Acid
ESCMID	European Society of Clinical Microbiology and Infectious Diseases
HAI	Healthcare-Associated Infection
HA-MRSA	Hospital-Acquired Methicillin-Resistant *Staphylococcus aureus*
ICA	Immunochromatographic Assay
ICU	Intensive Care Unit
LAMP	Loop-Mediated Isothermal Amplification
MIC	Minimum Inhibitory Concentration
MRSA	Methicillin-Resistant *Staphylococcus aureus*
PBP2a	Penicillin-Binding Protein 2a
PCR	Polymerase Chain Reaction
PPE	Personal Protective Equipment
PVL	Panton-Valentine Leukocidin
RNA	Ribonucleic Acid
SCCmec	Staphylococcal Cassette Chromosome mec
UTI	Urinary Tract Infection
VAP	Ventilator-Associated Pneumonia
VRE	Vancomycin-Resistant Enterococci
WHO	World Health Organization

## Appendix A

**Table A1:** Search strategy and Boolean logic applied across bibliographic databases and publisher platforms.

Database	Date of Last Search	Search String/Boolean Logic	Filters and Limits Applied	Records Retrieved (n)
PubMed	30 September 2025	((((“MRSA”[Title/Abstract]) OR (“methicillin-resistant Staphylococcus aureus”[MeSH Terms]) OR (“VRE”[Title/Abstract]) OR (“vancomycin-resistant Enterococcus”[MeSH Terms])) AND ((“nosocomial infection”[MeSH Terms]) OR (“hospital-acquired infection”[Title/Abstract]) OR (“healthcare-associated infection”[Title/Abstract]))) AND ((“infection control”[Title/Abstract]) OR (“antimicrobial resistance”[Title/Abstract]) OR (“clinical outcomes”[Title/Abstract]) OR (“diagnostic methods”[Title/Abstract]))))	Publication years: 2020–2025; Species: Humans; Language: English	38,967
Web of Science (Core Collection)	30 September 2025	TS = (“MRSA” OR “methicillin-resistant Staphylococcus aureus” OR “VRE” OR “vancomycin-resistant Enterococcus”) AND TS = (“nosocomial infection” OR “hospital-acquired infection” OR “HAI”) AND TS = (“infection control” OR “antimicrobial resistance” OR “clinical outcomes” OR “molecular diagnostics”)	Timespan: 2020–2025; Document type: Article; Language: English; Research areas: Infectious Diseases, Microbiology, Public Health	12,430
Scopus	30 September 2025	TITLE-ABS-KEY (“MRSA” OR “methicillin-resistant Staphylococcus aureus” OR “VRE” OR “vancomycin-resistant Enterococcus”) AND TITLE-ABS-KEY (“nosocomial infection” OR “hospital-acquired infection”) AND TITLE-ABS-KEY (“antimicrobial resistance” OR “infection control” OR “diagnostic methods”)	Years: 2020–2025; Language: English; Document type: Article	18,520
SpringerLink	30 September 2025	(“MRSA” OR “methicillin-resistant Staphylococcus aureus” OR “VRE” OR “vancomycin-resistant Enterococcus”) AND (“nosocomial infection” OR “hospital infection” OR “infection control”)	Content type: Journal articles; Years: 2020–2025	6780
ScienceDirect	30 September 2025	TITLE-ABS-KEY (“MRSA” OR “VRE” OR “multidrug-resistant Gram-positive cocci”) AND TITLE-ABS-KEY (“hospital-acquired infection” OR “nosocomial infection”) AND TITLE-ABS-KEY (“antimicrobial resistance” OR “clinical management” OR “diagnostic techniques”)	Years: 2020–2025; Language: English; Research domain: Medicine, Microbiology	7270
Wiley Online Library	30 September 2025	(“MRSA” OR “VRE”) AND (“healthcare-associated infection” OR “nosocomial infection”) AND (“molecular diagnostics” OR “screening strategies” OR “antimicrobial stewardship”)	Journals only; Years: 2020–2025; Language: English	2000
Frontiers (Frontiers in Microbiology/Frontiers in Public Health)	30 September 2025	(“MRSA” OR “methicillin-resistant Staphylococcus aureus” OR “VRE” OR “vancomycin-resistant Enterococcus”) AND (“nosocomial infection” OR “hospital-acquired infection” OR “healthcare-associated infection”) AND (“antimicrobial resistance” OR “infection control” OR “genomic epidemiology” OR “diagnostic methods”)	Years: 2020–2025; Article Type: Original Research; Language: English	3000
