# Correction: Peptine et al. Methicillin-Resistant *Staphylococcus aureus* (MRSA) and Vancomycin-Resistant Enterococci (VRE) in Nosocomial Infections: A Systematic Review of Resistance, Pathogenesis, and Clinical Management. *Microorganisms* 2026, *14*, 428

**DOI:** 10.3390/microorganisms14061258

**Published:** 2026-06-03

**Authors:** Lucian-Daniel Peptine, Andreea-Eliza Zaharia, Nicoleta-Maricica Maftei, Cosmin-Răducu Răileanu, Elena-Roxana Matache (Vasilache), Alice-Crina Conea, Bianca-Ioana Chesaru, Dana Tutunaru, Oana-Maria Dragostin, Liliana Mititelu-Tarţău, Gabriela Gurău

**Affiliations:** 1 Dunarea de Jos University of Galati, Faculty of Medicine and Pharmacy, Research Centre in the Medical-Pharmaceutical Field, 800008 Galati, Romania; lucian.peptine@ugal.ro (L.-D.P.); andreea.zaharia@ugal.ro (A.-E.Z.); elena.matache@ugal.ro (E.-R.M.); dr.alice.crina@gmail.com (A.-C.C.); bchesaru@ugal.ro (B.-I.C.); dana.tutunaru@ugal.ro (D.T.); oana.dragostin@ugal.ro (O.-M.D.); gabriela.gurau@ugal.ro (G.G.); 2 Sf. Ioan Emergency Clinical Hospital for Children, 80008 Galati, Romania; 3 Sf. Apostol Andrei County Emergency Clinical Hospital, 80008 Galati, Romania; 4 Grigore T. Popa University of Medicine and Pharmacy, 700115 Iasi, Romania; lylytartau@yahoo.com

In the original publication [1], there were mistakes in Figures 1 and 4 as published. They have been replaced with simplified schematic versions in order to improve graphical clarity and visual presentation. The corrected Figure 1 and Figure 4 appear below. In addition, several spelling errors in the main text were corrected.

The authors state that the scientific conclusions are unaffected. This correction was approved by the Academic Editor. The original publication has also been updated.

## Figures and Tables

**Figure 1:**
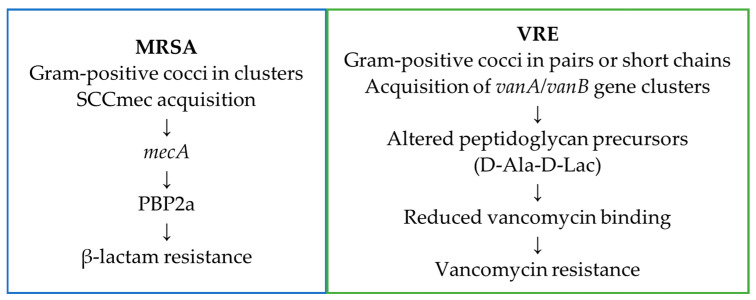
Simplified schematic representation of methicillin resistance in MRSA and vancomycin resistance in VRE. In MRSA, acquisition of the SCCmec element carrying *mecA* results in the expression of PBP2a and resistance to most β-lactam antibiotics. In VRE, *vanA* or *vanB* gene clusters result in the synthesis of altered peptidoglycan precursors terminating in D-Ala-D-Lac, thereby reducing vancomycin binding and conferring vancomycin resistance.

**Figure 4:**
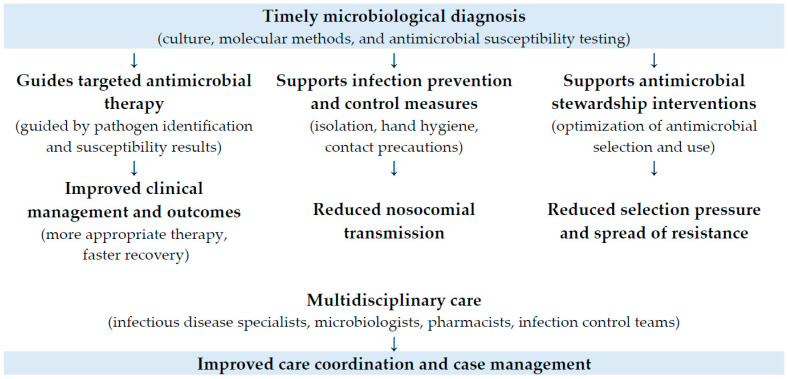
Conceptual overview of how timely microbiological diagnosis supports targeted antimicrobial therapy, infection prevention and control measures, antimicrobial stewardship, and multidisciplinary care, thereby contributing to improved clinical and institutional outcomes in nosocomial MRSA and VRE infections.
